# Aqua­bis(1*H*-imidazole-κ*N* ^3^)bis­(4-methyl­benzoato-κ^2^ *O*,*O*′)cadmium(II)

**DOI:** 10.1107/S1600536808011331

**Published:** 2008-04-26

**Authors:** Wen-Dong Song, Li-Li Ji, Xiang-Hu Huang

**Affiliations:** a College of Science, Guangdong Ocean University, Zhanjiang 524088, People’s Republic of China; b College of Fisheries, Guangdong Ocean University, ZhanJiang 524088, People’s Republic of China

## Abstract

In the title compound, [Cd(C_8_H_7_O_2_)_2_(C_3_H_4_N_2_)_2_(H_2_O)], the Cd^II^ atom is coordinated by four carboxyl­ate O atoms from two bidentate chelating 4-methyl­benzoate ligands, two N atoms from two imidazole ligands and one water mol­ecule in a distorted penta­gonal bipyramidal geometry. Inter­molecular O—H⋯O hydrogen bonds between the coordinated water mol­ecule and the carboxyl­ate O atoms of 4-methyl­benzoate lead to an infinite chain. These chains are further self-assembled into a two-dimensional layer through N—H⋯O hydrogen bonds between the imidazole ligands and carboxyl­ate groups. One of the imidazole ligands is disordered over two positions with site-occupancy factors of 0.737 (4) and 0.263 (4).

## Related literature

For related literature, see: Song *et al.* (2007 ▶).
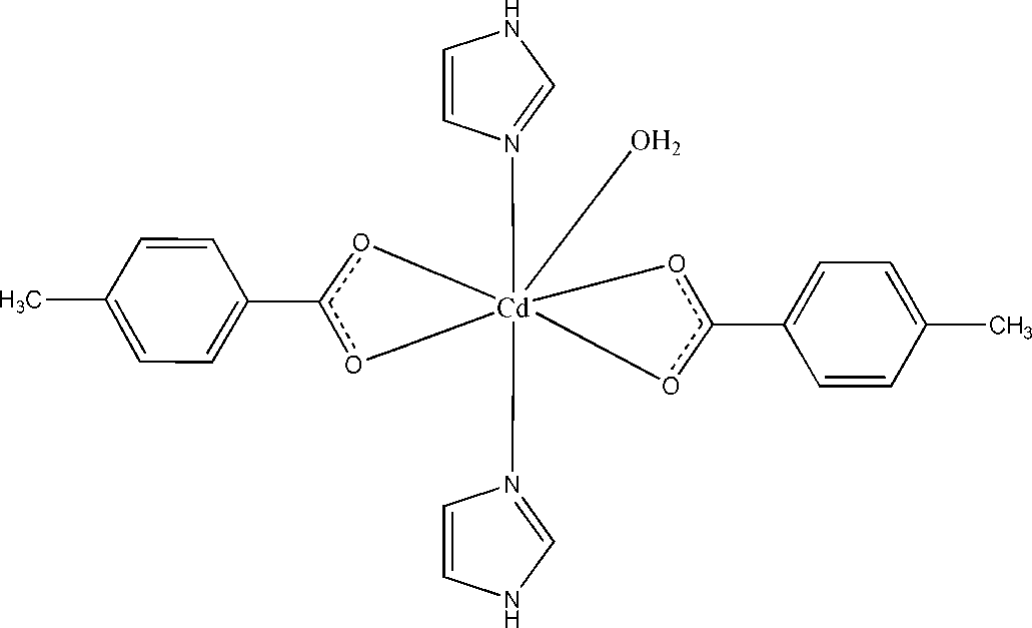


## Experimental

### 

#### Crystal data

[Cd(C_8_H_7_O_2_)_2_(C_3_H_4_N_2_)_2_(H_2_O)]
*M* *_r_* = 536.85
Triclinic, 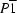

*a* = 6.1355 (1) Å
*b* = 12.4338 (3) Å
*c* = 15.4771 (3) Å
α = 91.396 (1)°
β = 97.765 (1)°
γ = 98.304 (1)°
*V* = 1156.46 (4) Å^3^
*Z* = 2
Mo *K*α radiation
μ = 0.98 mm^−1^
*T* = 296 (2) K
0.26 × 0.23 × 0.20 mm

#### Data collection

Bruker SMART APEXII CCD area-detector diffractometer
Absorption correction: multi-scan (*SADABS*; Sheldrick, 1996 ▶) *T*_min_ = 0.784, *T*_max_ = 0.828
14653 measured reflections
4764 independent reflections
4515 reflections with *I* > 2σ(*I*)
*R*_int_ = 0.037

#### Refinement

*R*[*F* ^2^ > 2σ(*F* ^2^)] = 0.023
*wR*(*F* ^2^) = 0.059
*S* = 1.08
4764 reflections
314 parameters
37 restraints
H atoms treated by a mixture of independent and constrained refinement
Δρ_max_ = 0.27 e Å^−3^
Δρ_min_ = −0.46 e Å^−3^

### 

Data collection: *APEX2* (Bruker, 2007 ▶); cell refinement: *SAINT* (Bruker, 2007 ▶); data reduction: *SAINT*; program(s) used to solve structure: *SHELXS97* (Sheldrick, 2008 ▶); program(s) used to refine structure: *SHELXL97* (Sheldrick, 2008 ▶); molecular graphics: *SHELXTL* (Sheldrick, 2008 ▶); software used to prepare material for publication: *SHELXTL*.

## Supplementary Material

Crystal structure: contains datablocks I, global. DOI: 10.1107/S1600536808011331/hy2128sup1.cif

Structure factors: contains datablocks I. DOI: 10.1107/S1600536808011331/hy2128Isup2.hkl

Additional supplementary materials:  crystallographic information; 3D view; checkCIF report

## Figures and Tables

**Table:** 

Cd1—N3	2.257 (13)
Cd1—N1	2.2805 (15)
Cd1—O1*W*	2.3514 (13)
Cd1—O4	2.3842 (16)
Cd1—O1	2.4128 (14)
Cd1—O2	2.4788 (15)
Cd1—O3	2.5507 (16)

**Table:** 

N3—Cd1—N1	174.2 (3)
N3—Cd1—O1*W*	85.9 (3)
N1—Cd1—O1*W*	88.49 (6)
N3—Cd1—O4	88.0 (5)
N1—Cd1—O4	92.50 (6)
O1*W*—Cd1—O4	81.32 (5)
N3—Cd1—O1	96.7 (5)
N1—Cd1—O1	86.88 (5)
O1*W*—Cd1—O1	141.36 (5)
O4—Cd1—O1	137.19 (5)
N3—Cd1—O2	88.6 (4)
N1—Cd1—O2	89.81 (5)
O1*W*—Cd1—O2	88.32 (5)
O4—Cd1—O2	169.31 (5)
O1—Cd1—O2	53.35 (5)
N3—Cd1—O3	89.8 (3)
N1—Cd1—O3	95.21 (5)
O1*W*—Cd1—O3	134.30 (5)
O4—Cd1—O3	53.05 (5)
O1—Cd1—O3	84.34 (5)
O2—Cd1—O3	137.09 (4)

**Table 2:** Hydrogen-bond geometry (Å, °)

*D*—H⋯*A*	*D*—H	H⋯*A*	*D*⋯*A*	*D*—H⋯*A*
O1*W*—H1*W*⋯O3^i^	0.82 (2)	2.104 (14)	2.881 (2)	157 (2)
O1*W*—H2*W*⋯O1^i^	0.82 (2)	1.943 (13)	2.723 (2)	160 (2)
N2—H2⋯O3^ii^	0.86	2.06	2.905 (3)	167
N4—H4*A*⋯O2^iii^	0.86	1.98	2.813 (8)	163

Symmetry codes: (i) 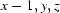; (ii) 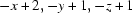; (iii) 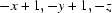.

**Fig. 1.**
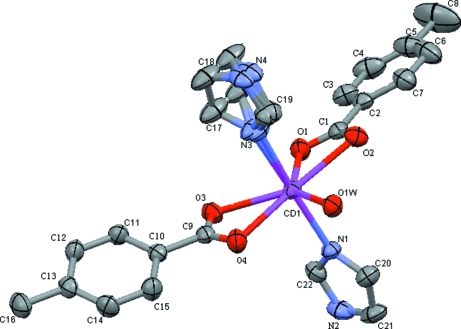
The molecular structure of the title compound. Displacement ellipsoids are drawn as the 30% probability level. H atoms have been omitted for clarity.

**Fig. 2.**
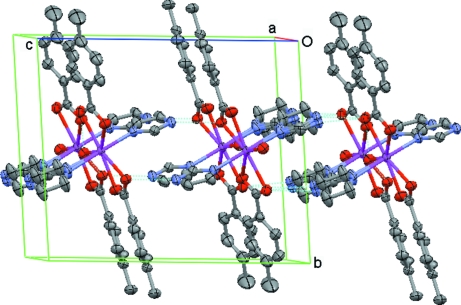
A view of the two-dimensional layer in the title compound.

## supplementary crystallographic information

### Comment

In the structural investigation of 4-methylbenzoate complexes, it has been found that the 4-methylbenzoate functions as bidentate and monodentate ligands (Song *et al.*, 2007). In this paper, we report the crystal structure of the title compound, a new Cd complex obtained by the reaction of 4-methylbenzoic acid, imidazole and cadmium chloride in alkaline aqueous solution.

As illustrated in Fig. 1, the Cd^II^ atom exists in a distorted pentagonal bipyramidal geometry, defined by four carboxylate O atoms from two bidentate chelating 4-methylbenzoate ligands, two N atoms from two imidazole ligands and one water molecule (Table 1). One of the imidazole ligands is disordered over two positions with site occupancy factors of 0.737 (4) and 0.263 (4). Intermolecular O—H···O hydrogen bonds (Table 2) lead to infinite chains, involving the coordinated water molecules as donors and the O atoms of 4-methylbenzoate ligands as acceptors. These chains are further self-assembled into a two-dimensional supramolecular network through intermolecular N—H···O hydrogen bonds between the imidazole ligands and carboxylate groups (Fig. 2).

### Experimental

A mixture of cadmium chloride (0.183 g, 1 mmol), 4-methylbenzoic acid (0.136 g, 1 mmol), imidazole (0.068 g, 1 mmol), NaOH (0.06 g, 1.5 mmol) and H_2_O (12 ml) was sealed in a 23 ml Teflon-lined reactor, which was heated at 433 K for 3 d and then cooled to room temperature at a rate of 10 K h^-1^. The crystals obtained were washed with water and dried in air.

### Refinement

H atoms attached to C and N atoms were positioned geometrically and refined as riding atoms, with C—H = 0.93 (aromatic) and 0.96 (methyl) Å and N—H = 0.86 Å and with *U*_iso_(H) = 1.2*U*_eq_(C,*N*). H atoms of the water molecule were located in a difference Fourier map and refined with restraints of O—H = 0.82 (1) Å and H···H= 1.34 (2) Å and with *U*_iso_(H) = 1.5*U*_eq_(O). Site occupancy factors of the disordered imidazole ligand were refined.

### Crystal data

**Table:**

[Cd(C_8_H_7_O_2_)_2_(C_3_H_4_N_2_)_2_(H_2_O)]	*Z* = 2
*M**_r_* = 536.85	*F*_000_ = 544
Triclinic, *P*1	*D*_x_ = 1.542 Mg m^−^^3^
Hall symbol: -P 1	Mo *K*α radiation λ = 0.71073 Å
*a* = 6.1355 (1) Å	Cell parameters from 3600 reflections
*b* = 12.4338 (3) Å	θ = 1.4–28º
*c* = 15.4771 (3) Å	µ = 0.98 mm^−^^1^
α = 91.396 (1)º	*T* = 296 (2) K
β = 97.765 (1)º	Block, colorless
γ = 98.304 (1)º	0.26 × 0.23 × 0.20 mm
*V* = 1156.46 (4) Å^3^	

### Data collection

**Table:**

Bruker SMART APEXII CCD area-detector diffractometer	4764 independent reflections
Radiation source: fine-focus sealed tube	4515 reflections with *I* > 2σ(*I*)
Monochromator: graphite	*R*_int_ = 0.037
*T* = 296(2) K	θ_max_ = 26.5º
φ and ω scans	θ_min_ = 1.3º
Absorption correction: multi-scan(SADABS; Sheldrick, 1996)	*h* = −7→7
*T*_min_ = 0.784, *T*_max_ = 0.828	*k* = −15→15
14653 measured reflections	*l* = −18→19

### Refinement

**Table:**

Refinement on *F*^2^	Secondary atom site location: difference Fourier map
Least-squares matrix: full	Hydrogen site location: inferred from neighbouring sites
*R*[*F*^2^ > 2σ(*F*^2^)] = 0.023	H atoms treated by a mixture of independent and constrained refinement
*wR*(*F*^2^) = 0.059	*w* = 1/[σ^2^(*F*_o_^2^) + (0.0265*P*)^2^ + 0.2332*P*] where *P* = (*F*_o_^2^ + 2*F*_c_^2^)/3
*S* = 1.08	(Δ/σ)_max_ = 0.010
4764 reflections	Δρ_max_ = 0.27 e Å^−^^3^
314 parameters	Δρ_min_ = −0.46 e Å^−^^3^
37 restraints	Extinction correction: SHELXTL (Sheldrick, 2008), Fc^*^=kFc[1+0.001xFc^2^λ^3^/sin(2θ)]^-1/4^
Primary atom site location: structure-invariant direct methods	Extinction coefficient: 0.0610 (16)

### Fractional atomic coordinates and isotropic or equivalent isotropic displacement parameters (Å^2^)

**Table:**

	*x*	*y*	*z*	*U*_iso_*/*U*_eq_	Occ. (<1)
C1	0.9109 (3)	0.68747 (15)	0.20150 (12)	0.0325 (4)	
C2	1.0359 (3)	0.79413 (16)	0.18052 (12)	0.0360 (4)	
C3	1.2641 (4)	0.81996 (19)	0.20736 (15)	0.0462 (5)	
H3	1.3405	0.7705	0.2383	0.055*	
C4	1.3774 (5)	0.9190 (2)	0.18814 (17)	0.0616 (7)	
H4	1.5296	0.9354	0.2065	0.074*	
C5	1.2686 (6)	0.9941 (2)	0.14216 (18)	0.0694 (9)	
C6	1.0429 (6)	0.9680 (2)	0.11549 (17)	0.0636 (7)	
H6	0.9671	1.0177	0.0844	0.076*	
C7	0.9270 (4)	0.86914 (19)	0.13418 (15)	0.0483 (5)	
H7	0.7749	0.8531	0.1154	0.058*	
C8	1.3923 (9)	1.1036 (3)	0.1231 (3)	0.1242 (18)	
H8A	1.4616	1.0964	0.0717	0.186*	
H8B	1.5041	1.1289	0.1717	0.186*	
H8C	1.2895	1.1549	0.1139	0.186*	
C9	0.7907 (3)	0.29695 (15)	0.32072 (12)	0.0339 (4)	
C10	0.8303 (3)	0.18454 (15)	0.34383 (12)	0.0327 (4)	
C11	1.0249 (3)	0.14673 (16)	0.33053 (14)	0.0393 (4)	
H11	1.1366	0.1925	0.3089	0.047*	
C12	1.0542 (4)	0.04120 (17)	0.34930 (15)	0.0422 (5)	
H12	1.1843	0.0165	0.3384	0.051*	
C13	0.8944 (4)	−0.02866 (16)	0.38390 (13)	0.0388 (4)	
C14	0.7012 (4)	0.01078 (17)	0.39875 (15)	0.0460 (5)	
H14	0.5927	−0.0338	0.4232	0.055*	
C15	0.6680 (4)	0.11496 (17)	0.37780 (15)	0.0429 (5)	
H15	0.5355	0.1389	0.3865	0.052*	
C16	0.9287 (5)	−0.14287 (18)	0.40544 (17)	0.0559 (6)	
H16A	1.0697	−0.1560	0.3905	0.084*	
H16B	0.8119	−0.1935	0.3728	0.084*	
H16C	0.9265	−0.1519	0.4667	0.084*	
C20	0.4871 (4)	0.61585 (18)	0.41076 (14)	0.0424 (5)	
H20	0.3509	0.6207	0.3777	0.051*	
C21	0.5421 (4)	0.64415 (19)	0.49653 (15)	0.0517 (6)	
H21	0.4521	0.6709	0.5332	0.062*	
C22	0.8214 (4)	0.58717 (17)	0.44726 (13)	0.0396 (4)	
H22	0.9619	0.5684	0.4451	0.047*	
Cd1	0.68370 (2)	0.491827 (10)	0.250662 (8)	0.02905 (7)	
N1	0.6629 (3)	0.57885 (13)	0.38005 (10)	0.0330 (3)	
N2	0.7550 (4)	0.62584 (15)	0.51870 (12)	0.0487 (5)	
H2	0.8326	0.6372	0.5695	0.058*	
O1	1.0144 (2)	0.62012 (12)	0.24245 (10)	0.0424 (3)	
O2	0.7032 (2)	0.66821 (12)	0.17868 (10)	0.0408 (3)	
O3	0.9568 (3)	0.36908 (11)	0.31565 (10)	0.0420 (3)	
O4	0.5945 (3)	0.31678 (12)	0.30732 (11)	0.0468 (4)	
O1W	0.2938 (2)	0.47353 (12)	0.22005 (10)	0.0395 (3)	
H1W	0.221 (4)	0.4298 (14)	0.2482 (15)	0.059*	
H2W	0.228 (4)	0.5262 (13)	0.2184 (17)	0.059*	
C17	0.8273 (6)	0.3438 (4)	0.0917 (2)	0.0580 (10)	0.737 (4)
H17	0.9576	0.3315	0.1257	0.070*	0.737 (4)
C18	0.7560 (6)	0.3045 (4)	0.0092 (2)	0.0633 (11)	0.737 (4)
H18	0.8312	0.2643	−0.0251	0.076*	0.737 (4)
C19	0.508 (3)	0.3921 (12)	0.0535 (6)	0.0420 (16)	0.737 (4)
H19	0.3728	0.4179	0.0538	0.050*	0.737 (4)
N3	0.668 (2)	0.4081 (12)	0.1184 (10)	0.0411 (13)	0.737 (4)
N4	0.5551 (7)	0.3347 (5)	−0.0138 (5)	0.0450 (11)	0.737 (4)
H4A	0.4719	0.3196	−0.0632	0.054*	0.737 (4)
C17'	0.8520 (17)	0.4185 (11)	0.0683 (6)	0.0580 (10)	0.263 (4)
H17'	0.9919	0.4571	0.0883	0.070*	0.263 (4)
C18'	0.7834 (18)	0.3789 (12)	−0.0139 (6)	0.0633 (11)	0.263 (4)
H18'	0.8712	0.3675	−0.0568	0.076*	0.263 (4)
C19'	0.512 (7)	0.379 (4)	0.0592 (19)	0.0420 (16)	0.263 (4)
H19'	0.3690	0.3811	0.0721	0.050*	0.263 (4)
N3'	0.690 (7)	0.394 (4)	0.117 (3)	0.0411 (13)	0.263 (4)
N4'	0.561 (2)	0.3591 (18)	−0.0207 (18)	0.0450 (11)	0.263 (4)
H4'	0.4683	0.3381	−0.0670	0.054*	0.263 (4)

### Atomic displacement parameters (Å^2^)

**Table:**

	*U*^11^	*U*^22^	*U*^33^	*U*^12^	*U*^13^	*U*^23^
C1	0.0379 (10)	0.0348 (10)	0.0259 (9)	0.0070 (8)	0.0076 (8)	−0.0024 (7)
C2	0.0439 (11)	0.0367 (10)	0.0281 (9)	0.0036 (8)	0.0108 (8)	−0.0024 (8)
C3	0.0432 (11)	0.0526 (13)	0.0419 (12)	0.0010 (10)	0.0103 (9)	−0.0041 (10)
C4	0.0575 (15)	0.0704 (17)	0.0511 (14)	−0.0175 (13)	0.0183 (12)	−0.0110 (13)
C5	0.097 (2)	0.0552 (15)	0.0481 (15)	−0.0246 (15)	0.0208 (15)	−0.0025 (12)
C6	0.095 (2)	0.0448 (13)	0.0475 (14)	0.0013 (14)	0.0059 (14)	0.0121 (11)
C7	0.0587 (14)	0.0435 (12)	0.0412 (12)	0.0043 (10)	0.0039 (10)	0.0052 (10)
C8	0.179 (5)	0.079 (2)	0.092 (3)	−0.065 (3)	0.025 (3)	0.011 (2)
C9	0.0439 (11)	0.0332 (10)	0.0256 (9)	0.0082 (8)	0.0060 (8)	0.0000 (7)
C10	0.0386 (10)	0.0293 (9)	0.0291 (9)	0.0033 (7)	0.0032 (8)	−0.0003 (7)
C11	0.0404 (11)	0.0339 (10)	0.0454 (12)	0.0041 (8)	0.0138 (9)	0.0038 (8)
C12	0.0431 (11)	0.0362 (11)	0.0506 (13)	0.0127 (9)	0.0113 (10)	0.0007 (9)
C13	0.0500 (12)	0.0281 (9)	0.0363 (10)	0.0036 (8)	0.0023 (9)	−0.0014 (8)
C14	0.0474 (12)	0.0357 (11)	0.0548 (13)	−0.0016 (9)	0.0135 (10)	0.0081 (9)
C15	0.0363 (10)	0.0381 (11)	0.0554 (13)	0.0048 (8)	0.0108 (9)	0.0032 (9)
C16	0.0824 (18)	0.0313 (11)	0.0546 (14)	0.0106 (11)	0.0091 (13)	0.0045 (10)
C20	0.0400 (11)	0.0468 (12)	0.0405 (11)	0.0038 (9)	0.0100 (9)	−0.0049 (9)
C21	0.0681 (16)	0.0471 (12)	0.0410 (12)	−0.0028 (11)	0.0259 (12)	−0.0112 (10)
C22	0.0473 (11)	0.0346 (10)	0.0341 (11)	0.0025 (8)	−0.0010 (9)	0.0037 (8)
Cd1	0.03054 (9)	0.03191 (10)	0.02451 (9)	0.00588 (5)	0.00252 (5)	−0.00215 (5)
N1	0.0391 (8)	0.0324 (8)	0.0271 (8)	0.0045 (6)	0.0050 (7)	−0.0009 (6)
N2	0.0710 (14)	0.0416 (10)	0.0264 (9)	−0.0088 (9)	0.0009 (8)	−0.0006 (7)
O1	0.0410 (8)	0.0393 (8)	0.0496 (9)	0.0120 (6)	0.0089 (7)	0.0074 (6)
O2	0.0381 (8)	0.0463 (8)	0.0358 (8)	0.0026 (6)	0.0008 (6)	0.0045 (6)
O3	0.0522 (9)	0.0313 (7)	0.0417 (8)	0.0018 (6)	0.0085 (7)	0.0054 (6)
O4	0.0481 (9)	0.0409 (8)	0.0545 (10)	0.0159 (7)	0.0080 (7)	0.0059 (7)
O1W	0.0303 (7)	0.0441 (8)	0.0447 (8)	0.0086 (6)	0.0041 (6)	0.0038 (7)
C17	0.0427 (15)	0.089 (3)	0.0413 (17)	0.0138 (19)	0.0016 (13)	−0.0240 (18)
C18	0.0542 (18)	0.092 (3)	0.0433 (18)	0.013 (2)	0.0090 (14)	−0.0268 (19)
C19	0.0507 (14)	0.040 (4)	0.0335 (17)	0.008 (2)	−0.0013 (14)	0.001 (2)
N3	0.040 (3)	0.053 (4)	0.0312 (11)	0.0127 (18)	0.0034 (19)	−0.013 (2)
N4	0.0624 (13)	0.041 (3)	0.0262 (17)	−0.0024 (13)	−0.0035 (9)	−0.002 (2)
C17'	0.0427 (15)	0.089 (3)	0.0413 (17)	0.0138 (19)	0.0016 (13)	−0.0240 (18)
C18'	0.0542 (18)	0.092 (3)	0.0433 (18)	0.013 (2)	0.0090 (14)	−0.0268 (19)
C19'	0.0507 (14)	0.040 (4)	0.0335 (17)	0.008 (2)	−0.0013 (14)	0.001 (2)
N3'	0.040 (3)	0.053 (4)	0.0312 (11)	0.0127 (18)	0.0034 (19)	−0.013 (2)
N4'	0.0624 (13)	0.041 (3)	0.0262 (17)	−0.0024 (13)	−0.0035 (9)	−0.002 (2)

### Geometric parameters (Å, °)

**Table:**

C1—O1	1.256 (2)	C20—N1	1.369 (3)
C1—O2	1.262 (3)	C20—H20	0.9300
C1—C2	1.499 (3)	C21—N2	1.358 (3)
C2—C7	1.383 (3)	C21—H21	0.9300
C2—C3	1.393 (3)	C22—N1	1.315 (3)
C3—C4	1.384 (3)	C22—N2	1.331 (3)
C3—H3	0.9300	C22—H22	0.9300
C4—C5	1.381 (5)	Cd1—N3	2.257 (13)
C4—H4	0.9300	Cd1—N1	2.2805 (15)
C5—C6	1.379 (5)	Cd1—O1W	2.3514 (13)
C5—C8	1.516 (4)	Cd1—O4	2.3842 (16)
C6—C7	1.386 (3)	Cd1—O1	2.4128 (14)
C6—H6	0.9300	Cd1—O2	2.4788 (15)
C7—H7	0.9300	Cd1—O3	2.5507 (16)
C8—H8A	0.9600	N2—H2	0.8600
C8—H8B	0.9600	O1W—H1W	0.82 (2)
C8—H8C	0.9600	O1W—H2W	0.82 (2)
C9—O4	1.254 (3)	C17—C18	1.347 (4)
C9—O3	1.269 (2)	C17—N3	1.444 (10)
C9—C10	1.496 (3)	C17—H17	0.9300
C10—C11	1.383 (3)	C18—N4	1.345 (5)
C10—C15	1.390 (3)	C18—H18	0.9300
C11—C12	1.382 (3)	C19—N3	1.298 (4)
C11—H11	0.9300	C19—N4	1.337 (6)
C12—C13	1.386 (3)	C19—H19	0.9300
C12—H12	0.9300	N4—H4A	0.8600
C13—C14	1.391 (3)	C17'—N3'	1.33 (5)
C13—C16	1.504 (3)	C17'—C18'	1.343 (8)
C14—C15	1.380 (3)	C17'—H17'	0.9300
C14—H14	0.9300	C18'—N4'	1.343 (9)
C15—H15	0.9300	C18'—H18'	0.9300
C16—H16A	0.9600	C19'—N3'	1.300 (9)
C16—H16B	0.9600	C19'—N4'	1.337 (10)
C16—H16C	0.9600	C19'—H19'	0.9300
C20—C21	1.350 (3)	N4'—H4'	0.8600
			
O1—C1—O2	121.53 (18)	N3—Cd1—O1	96.7 (5)
O1—C1—C2	119.37 (18)	N1—Cd1—O1	86.88 (5)
O2—C1—C2	119.09 (18)	O1W—Cd1—O1	141.36 (5)
C7—C2—C3	118.6 (2)	O4—Cd1—O1	137.19 (5)
C7—C2—C1	120.66 (19)	N3—Cd1—O2	88.6 (4)
C3—C2—C1	120.8 (2)	N1—Cd1—O2	89.81 (5)
C4—C3—C2	120.2 (2)	O1W—Cd1—O2	88.32 (5)
C4—C3—H3	119.9	O4—Cd1—O2	169.31 (5)
C2—C3—H3	119.9	O1—Cd1—O2	53.35 (5)
C5—C4—C3	121.2 (3)	N3—Cd1—O3	89.8 (3)
C5—C4—H4	119.4	N1—Cd1—O3	95.21 (5)
C3—C4—H4	119.4	O1W—Cd1—O3	134.30 (5)
C6—C5—C4	118.4 (2)	O4—Cd1—O3	53.05 (5)
C6—C5—C8	120.6 (4)	O1—Cd1—O3	84.34 (5)
C4—C5—C8	121.0 (3)	O2—Cd1—O3	137.09 (4)
C5—C6—C7	121.1 (3)	C22—N1—C20	105.43 (17)
C5—C6—H6	119.4	C22—N1—Cd1	123.23 (14)
C7—C6—H6	119.4	C20—N1—Cd1	130.55 (14)
C2—C7—C6	120.5 (2)	C22—N2—C21	107.39 (19)
C2—C7—H7	119.8	C22—N2—H2	126.3
C6—C7—H7	119.8	C21—N2—H2	126.3
O4—C9—O3	122.22 (19)	C1—O1—Cd1	94.15 (12)
O4—C9—C10	118.90 (18)	C1—O2—Cd1	90.92 (12)
O3—C9—C10	118.88 (18)	C9—O3—Cd1	87.86 (12)
C11—C10—C15	118.59 (18)	C9—O4—Cd1	95.91 (13)
C11—C10—C9	121.38 (17)	Cd1—O1W—H1W	117.5 (18)
C15—C10—C9	120.02 (18)	Cd1—O1W—H2W	121.9 (18)
C10—C11—C12	120.31 (18)	H1W—O1W—H2W	103.8 (15)
C10—C11—H11	119.8	C18—C17—N3	108.7 (6)
C12—C11—H11	119.8	C18—C17—H17	125.6
C11—C12—C13	121.63 (19)	N3—C17—H17	125.6
C11—C12—H12	119.2	C17—C18—N4	106.7 (5)
C13—C12—H12	119.2	C17—C18—H18	126.6
C12—C13—C14	117.65 (19)	N4—C18—H18	126.6
C12—C13—C16	121.4 (2)	N3—C19—N4	113.3 (12)
C14—C13—C16	121.0 (2)	N3—C19—H19	123.3
C15—C14—C13	121.05 (19)	N4—C19—H19	123.3
C15—C14—H14	119.5	C19—N3—C17	102.8 (11)
C13—C14—H14	119.5	C19—N3—Cd1	130.1 (8)
C14—C15—C10	120.7 (2)	C17—N3—Cd1	126.5 (8)
C14—C15—H15	119.6	C19—N4—C18	108.0 (9)
C10—C15—H15	119.6	C19—N4—H4A	126.0
C21—C20—N1	109.4 (2)	C18—N4—H4A	126.0
C21—C20—H20	125.3	N3'—C17'—C18'	110 (2)
N1—C20—H20	125.3	N3'—C17'—H17'	125.1
C20—C21—N2	106.33 (19)	C18'—C17'—H17'	125.1
C20—C21—H21	126.8	C17'—C18'—N4'	105.1 (14)
N2—C21—H21	126.8	C17'—C18'—H18'	127.5
N1—C22—N2	111.4 (2)	N4'—C18'—H18'	127.5
N1—C22—H22	124.3	N3'—C19'—N4'	111 (4)
N2—C22—H22	124.3	N3'—C19'—H19'	124.3
N3—Cd1—N1	174.2 (3)	N4'—C19'—H19'	124.3
N3—Cd1—O1W	85.9 (3)	C19'—N3'—C17'	103 (4)
N1—Cd1—O1W	88.49 (6)	C19'—N4'—C18'	106 (3)
N3—Cd1—O4	88.0 (5)	C19'—N4'—H4'	127.1
N1—Cd1—O4	92.50 (6)	C18'—N4'—H4'	127.1
O1W—Cd1—O4	81.32 (5)		

### Hydrogen-bond geometry (Å, °)

**Table:**

*D*—H···*A*	*D*—H	H···*A*	*D*···*A*	*D*—H···*A*
O1W—H1W···O3^i^	0.82 (2)	2.104 (14)	2.881 (2)	157 (2)
O1W—H2W···O1^i^	0.82 (2)	1.943 (13)	2.723 (2)	160 (2)
N2—H2···O3^ii^	0.86	2.06	2.905 (3)	167
N4—H4A···O2^iii^	0.86	1.98	2.813 (8)	163

Symmetry codes: (i) *x*−1, *y*, *z*; (ii) −*x*+2, −*y*+1, −*z*+1; (iii) −*x*+1, −*y*+1, −*z*.
